# A case report of *Mycoplasma wenyonii* associated immune-mediated haemolytic anaemia in a dairy cow

**DOI:** 10.1186/s13620-016-0061-x

**Published:** 2016-01-22

**Authors:** Nicola Gladden, Hayley Haining, Livia Henderson, Francesco Marchesi, Libby Graham, Michael McDonald, Fraser. R. Murdoch, Anna Bruguera Sala, Jayne Orr, Kathryn Ellis

**Affiliations:** 1 Scottish Centre for Production Animal Health and Food Safety, School of Veterinary Medicine, College of Medicine, Veterinary and Life Sciences, University of Glasgow, Garscube Campus, Bearsden Road, Glasgow, G61 1QH UK; 2 Veterinary Diagnostic Services, School of Veterinary Medicine, College of Medicine, Veterinary and Life Sciences, University of Glasgow, Garscube Campus, Bearsden Road, Glasgow, G61 1QH UK; 3 Royal (Dick) School of Veterinary Studies, University of Edinburgh, Easter Bush Campus, Edinburgh, Midlothian EH25 9RG UK

**Keywords:** Bovine, Immune-mediated haemolytic anaemia, *Mycoplasma wenyonii*

## Abstract

**Background and case presentation:**

A three year old, second lactation Holstein dairy cow presented to the Scottish Centre for Production Animal Health and Food Safety, Glasgow University Veterinary School in November 2014 with a history of post-calving vulval/vaginal bleeding nine days prior to presentation, followed by a sudden reduction in milk yield. Subsequent investigations resulted in a diagnosis of immune-mediated haemolytic anaemia secondary to infection with *Mycoplasma wenyonii*.

**Conclusion:**

This report of a novel presentation of *Mycoplasma wenyonii* in a dairy cow illustrates the need to consider *M.wenyonii* as a potential differential diagnosis when a cow presents with anaemia and will discuss the potential implications of the condition at herd-level.

## Background

Haemotropic mycoplasmas (haemoplasmas) associate with erythrocytes and are known to cause both acute and chronic disease in a number of species. Two haemotropic mycoplasmas are known to affect cattle: *Mycoplasma wenyonii* (formerly *Eperythrozoon wenyonii*) and the recently discovered *Candidatus* Mycoplasma haemobos. The association between *Mycoplasma haemofelis* (formerly *Haemobartonella felis*) and haemolytic anaemia is well documented in cats and a positive direct Coombs’ test result associated with *M.haemofelis* infection has been reported [1]; however, to our knowledge this association has not been reported in cattle infected with *M.wenyonii*.

Reports of anaemia in the absence of other more typical clinical signs, such as hind limb and udder or scrotal oedema, are infrequent in cases of *Mycoplasma wenyonii* infection in mature cattle [2, 3]. This report of a novel presentation of *Mycoplasma wenyonii* in a dairy cow illustrates the need to consider *M.wenyonii* as a potential differential diagnosis when presented with a cow with anaemia.

## Case presentation

A three year old, second lactation Holstein dairy cow presented to the Scottish Centre for Production Animal Health and Food Safety (SCPAHFS), School of Veterinary Medicine, University of Glasgow in November 2014 with a history of post-calving vulval/vaginal bleeding nine days prior to presentation followed by a sudden cessation in milk production. The animal was referred from a 230 cow, year round calving, closed dairy herd in Dumfries and Galloway, south-west Scotland. Cattle are housed all year round and fed a total mixed ration *ad libitum*. Animals receive leptospirosis, salmonella, infectious bovine rhinotracheitis (IBR) and bovine viral diarrhoea (BVD) vaccinations and the herd is a BVD negative herd in accordance with the Scottish government BVD eradication scheme. All cows are treated in the summer with a deltamethrin pour-on product (Butox Swish, MSD Animal Health, Milton Keynes, UK). This cow was homebred and had not left the farm during its lifetime until it was referred into the veterinary school.

On admission the animal was bright, alert and responsive and had a normal appetite and thirst. Tachycardia (100 beats/min) was evident on clinical examination but temperature and respiratory rate were within normal limits. Pallor of oral, conjunctival and vaginal mucous membranes was evident, raising a clinical suspicion of anaemia. There was no vaginal discharge. The remainder of the clinical examination was unremarkable.

History and initial clinical examination enabled significant calving trauma, ongoing haemorrhage and toxicities to be ruled out as potential differential diagnoses for the suspected anaemia in this animal prior to further investigation.

### Investigations

**Fig. 1:**
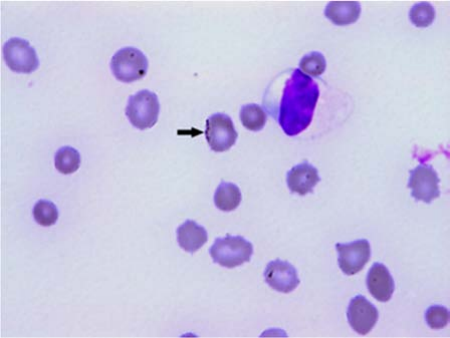
Blood smear (May-Grünwald-Giemsa x1000). Arrow indicates inclusion bodies on erythrocyte surface

**Fig. 2:**
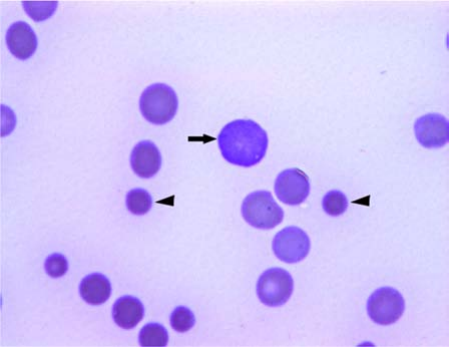
Blood smear (May-Grünwald-Giemsa x1000). Polychromatic erythrocyte (arrow) and spherocytes (arrow heads)

An EDTA-anticoagulated blood sample taken on admission revealed a markedly regenerative, severe (haematocrit 9.9 %; ref: 24-46 %) macrocytic anaemia with spherocytes and basophilic stippling present on the blood smear (Figs. 1 and 2) (Table 1). Serum biochemistry revealed hyperbilirubinaemia and elevated alkaline phosphatase (ALKP), aspartate aminotransferase (AST), gamma-glutamyl transferase (GGT) and glutamate dehydrogenase (GLDH) (Table 2), remaining serum biochemical parameters were all within the reference range. These biochemical abnormalities, together with the presence of a regenerative anaemia raised the suspicion of a haemolytic anaemia.

**Table 1:** Haematological parameters

Date	19/11/14	21/11/14	25/11/14	3/12/14	8/12/14	16/12/14
Haematocrit (%) Ref 24-46 %	9.9	15.2	19.8	22.8	22.2	22.1
Erythrocyte Count (x10^12^/l) Ref: 5-10x10^12^/l	1.02	1.52	2.24	3.3	3.48	3.8
Haemoglobin (g/dl) Ref: 8-15 g/dl	2.8	4.2	5.9	7.5	7.2	7.2
Mean Corpuscular Volume (fl) Ref: 40-60	96.7	99.9	19.8	69.2	63.7	58
Red Cell Distribution Width (%)	29.9	25.6	22.2	30.8	34.7	38.6
Blood smear	Spherocytes Inclusion bodies	Spherocytes, acanthocytes and schistocytes.	Spherocytes, acanthocytes and schistocytes. Basophilic inclusion bodies still observed	Small numbers of acanthocytes schistocytes and keratocytes present. Small number of inclusions still present.	Small numbers of acanthocytes schistocytes and keratocytes present. Small number of inclusions still present.	Occasional small inclusions seen on a very few erythrocytes. Can no longer confirm ongoing bacteraemia from smear examination only.
PCR result	POSITIVE				NEGATIVE	POSITIVE

**Table 2:** Biochemical parameters

Date	19/11/14	21/11/14	25/11/14	3/12/14	8/12/14	16/12/14
Total Bilirubin (μmol/l) Ref: <8	23	6	6	4	4	2
Alkaline Phosphatase (U/l) Ref: 20-285	385	268	184	95	98	92
Aspartate Aminotransferase (U/l) Ref: <140	283	173	127	62	37	42
Gamma-Glutamyl Transferase (U/l) Ref: <27	88	66	51	28	27	20
Glutamate Dehydrogenase (U/l) Ref: <10	190	23	10	8	4	6

Haemolytic anaemias commonly result in elevated liver enzymes and increased total bilirubin. Pathologic haemolysis increases haemoglobin degradation and consequent bilirubin formation exceeds the hepatic capacity for uptake and conjugation of bilirubin and excretion into bile, resulting in bilirubinaemia. Upregulation of hepatic function in response to increased need for bilirubin excretion results in increases in liver enzymes seen on serum biochemistry in cases of haemolytic anaemia.

There was no evidence of haemoglobinuria or haematuria on either urine dipstick analysis or laboratory urinalysis.

The presence of inclusion bodies on the blood smear coupled with a positive direct Coombs’ test meant a diagnosis of immune-mediated haemolytic anaemia could be made with suspected underlying infectious aetiology. The inclusion bodies seen on the blood smear were confirmed to be *Mycoplasma wenyonii* parasites by polymerase chain reaction (PCR).

Genus-specific *Mycoplasma* primers [4, 5] generated a specific 270 bp product from whole blood DNA. The Sanger-sequenced product (Source BioScience, Lanarkshire, UK) showed high sequence similarity to *Mycoplasma wenyonii* str. Massachusetts (NCBI GenBank accession number CP003703 [6] with 97.8 % (forward primer) and 98.7 % (reverse primer) probability.

### Treatment

There is a paucity of information available regarding the treatment of either haemotropic mycoplasmas or immune mediated haemolytic anaemia in cattle. Tetracyclines have been reported to be effective in treating both *M.haemofelis* and *M.suis* infections [7, 8] and there are reports of *M.wenyonii* in cattle being treated with oxytetracycline, however response to treatment has been variable [3, 9, 10]. In light of this, oxytetracycline was the treatment of choice in this case. Corticosteroids were considered, as part of this clinical presentation was immune-mediated; however, previous work has shown that Coombs’ positive cats infected with *M.haemofelis* respond to antibiotic treatment alone [1]. In addition to this, there are some cases where concurrent corticosteroid therapy has been shown to delay the clearance of haemotropic *Mycoplasma* from the blood [7]. It was decided to treat with oxytetracycline alone initially and only use concurrent corticosteroid if warranted by deteriorating clinical status of the animal in spite of appropriate antibiotic therapy. In light of the degree of anaemia exhibited by this animal blood transfusion was considered, however due to the clinical stability of the cow and concerns regarding the possibility of adverse transfusion reactions it was elected not to perform a blood transfusion on this occasion.

### Case progression and follow up

The cow was treated with 20 mg/kg long-acting oxytetracycline (Alamycin LA 300; Norbrook, Newry, Northern Ireland, UK) every three days by deep intramuscular injection. A blood sample was repeated 48 h following the commencement of treatment which showed a good initial response; haematocrit increased from 9.9 % to 15.2 % (Table 1), and total bilirubin reduced from 23 μmol/l to 6 μmol/l (Table 2). In addition, all elevated liver enzymes were reduced (Table 2). The cow initially improved in demeanour and the mucous membrane pallor resolved as the haematocrit improved, which was to be expected.

Blood samples for haematology and biochemistry were repeated regularly for the duration of treatment. The haematological improvement continued for the first week following treatment, from which point no further improvement in haematological parameters occurred despite continued treatment (Table 1). Liver enzymes and total bilirubin showed rapid initial improvement then remained within the reference range for the duration of treatment (Table 2). Blood taken on the 8^th^ of December 2014 was PCR negative for *Mycoplasma* species however there were still a few inclusion bodies seen in small numbers of erythrocytes, which were suggestive of continued *Mycoplasma* species infection in spite of a negative PCR result. A direct Coomb’s test was now negative.

After an initial improvement in demeanour and haematological and biochemical parameters, the cow failed to improve further and approximately three weeks after admission started to deteriorate in demeanour with the development of a soft cough and a persistent tachycardia (heart rate persistently greater than 90 beats per minute). The decision was made to euthanase the cow on the 19^th^ of December 2014, one month after presentation to the SCPAHFS due to limited further improvement and because the prognosis was considered to ultimately be poor.

A blood sample was taken immediately prior to euthanasia and submitted for repeat PCR testing. This blood sample was positive on PCR for *Mycoplasma* species however, this could not be confirmed to be *M.wenyonii* on sequence analysis.

### Post-mortem findings

**Fig. 3:**
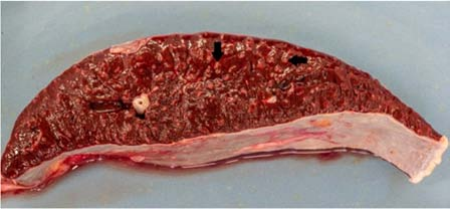
Spleen cut surface - the red pulp has a diffuse brick red to brownish discolouration and there is an increased prominence of lymphoid follicles in the white pulp (arrows)

Post mortem examination showed mild hepatomegaly, mild enlargement of the spleen with prominent lymphoid follicles within the white pulp (Fig. 3) and mild lymphadenomegaly of the right pre-scapular lymph node. Samples of spleen, lymph node, liver and bone marrow were collected in fixative (10 % neutral buffered formalin) and processed for paraffin embedding and sectioning for histological examination. Sections were stained with Haematoxylin and Eosin (H & E). An additional section of liver was stained with Perl’s Prussian Blue.

**Fig. 4:**
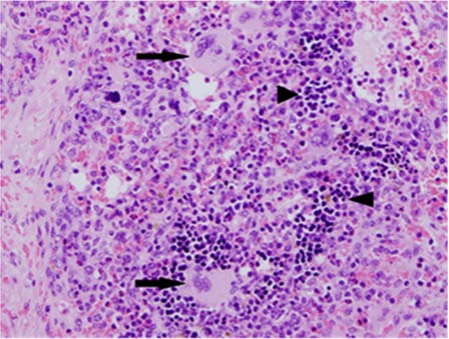
H&E section of lymph node showing EMH (this is an unusual location to find EMH). Both megakaryocytes (arrows) and nucleated erythroid precursors (arrowheads can be seen in clusters within the medullary cords

**Fig. 5:**
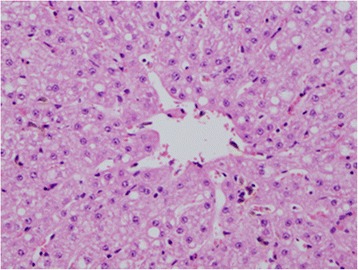
H&E section of liver showing yellowish-brownish granules in the Kupffer cells

**Fig. 6:**
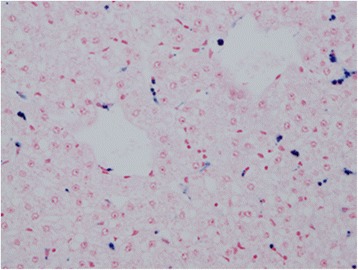
Perl’s Prussian Blue staining showing haemosiderin pigments in the Kupffer cells (detected as a blue stain within Kupffer cells)

Histological examination of the spleen confirmed expansion of the lymphoid follicles with prominent germinal centres within the white pulp. The red pulp was moderately congested with an increase in haemosiderin-laden macrophages. In the right pre-scapular lymph node, there was an increase in size and number of lymphoid follicles with expanded germinal centres, along with congestion of the paratrabecular and medullary sinuses. Numerous aggregates of nucleated erythroid precursors and variable numbers of megakaryocytes were noted in the medullary cords (Fig. 4). Hemosiderin-laden macrophages were also prominent in the lymph node. Large numbers of erythroid precursors and megakaryocytes were noted in a section from a bone marrow sample. Increased intracytoplasmic accumulation of haemosiderin (confirmed by Perl’s Prussian Blue staining) was also noted in Kupffer cells in hepatic sinusoids (Figs. 5 and 6).

Increased haemosiderin accumulation in the spleen, lymph node and liver is consistent with increased erythrocyte turnover as a result of extravascular haemolysis in the context of haemolytic anaemia. The occurrence of extramedullary haematopoiesis (EMH) in the lymph node, characterised by a predominance of erythroid and megakaryocytic elements and the apparent increase in erythroid precursors and megakaryocytes in the bone marrow are both consistent with a regenerative response secondary to anaemia.

## Discussion

Haemolytic anaemia is not common in cattle [11, 12] although parasitic anaemia occurs more commonly, particularly in tropical regions. *Babesia*, *Anaplasma*, *Theileria and Mycoplasma* species have all been reported to cause anaemia in cattle [13, 14]*.* Haemotropic *Mycoplasma* species have also been reported to cause haemolytic anaemia in small ruminants [15, 16] and pigs [8].

Initially haemorrhage secondary to significant calving trauma was suspected to be the most likely cause of anaemia in this case due to the history of vulval/vaginal bleeding soon after calving. This however was ruled out based on clinical examination and the history form the farmer reporting that the cow calved unassisted. Further support of the belief that acute haemorrhage was an unlikely cause of anaemia in this case was the stable clinical appearance of the animal in spite of a very low PCV which would suggest a more chronic, insidious disease progression (allowing the animal to establish compensatory mechanisms). Post-parturient haemoglobinuria was also considered as a potential differential diagnosis but phosphorus levels were within the reference range. Abomasal haemorrhage was ruled out based on the absence of melena and bright, appetant demeanour of the animal with no apparent abdominal pain. Post-mortem examination further confirmed that neither abomasal haemorrhage nor ulceration were evident in this animal.

Haematology and serum biochemistry indicated that the anaemia in this case was haemolytic and other potential causes of haemolytic anaemia were investigated. Heavy metal toxicities and adverse drug reactions have been reported to cause haemolytic anaemia in cattle [11, 17, 18] but could be ruled out in this case by the histories from the referring veterinary surgeon and the farmer. Similarly, haemolytic anaemias associated with onion, *brassica* and bracken toxicities were ruled out by history and laboratory findings.

Other parasitic and infectious causes of haemolytic anaemia such as babesiosis, anaplasmosis and leptospirosis were also considered as potential underlying differential diagnoses. Leptospirosis was considered to be very unlikely as this animal (and the herd from which it was referred) was vaccinated with a leptospirosis vaccine and there was no evidence in the history to suggest that this had been ineffectively instigated. This animal presented in November, which in Scotland makes tick borne infections such as anaplasmosis and babesiosis unlikely due to the environmental conditions at that time of year; in addition this cow was housed all year round therefore tick exposure was much less likely in this case.

Haemotropic mycoplasmas have been reported in a number of domestic species, as well as humans and are known to cause both chronic and acute disease [19]. Haemotropic *Mycoplasma* species adhere to the erythrocyte cell wall and cause haemolysis, resulting in anaemia. The exact mechanism resulting in haemolytic anaemia is not yet fully understood, however several mechanisms have been proposed including direct damage to the erythrocyte cell wall and the development of autoantibodies resulting in an immune-mediated anaemia [19]. In sheep, infection with *M.ovis* has been shown to increase the fragility of erythrocytes compared to PCR-negative control animals [20]. Autoreactive actin antibodies have been identified in pigs infected with *M.suis* and it is likely that these are involved in lysis of infected erythrocytes [8]. Autoantibodies have also been reported in haemotropic *Mycoplasma* infections in other species including cats, dogs, mice and rats [21–23]. To our knowledge the presence of autoantibodies has not yet been identified in cattle infected with *M.wenyonii* however it has been speculated that the hind limb oedema more commonly seen associated with *M.wenyonii* infections in cattle could result from the formation of local immune-complexes [9]. In cats the association between *Mycoplasma haemofelis* and positive direct Coombs’ test has been well documented [1, 7]; however, to our knowledge there are no reports in the literature of this association occurring in cattle, indeed there are relatively few cases of immune-mediated haemolytic anaemia of any cause reported in cattle [11, 12].

Disease caused by *Mycoplasma wenyonii* typically presents in individual animals as reduced milk yield and pitting oedema of the hind limbs and udder or scrotum. In the animal described in this case study, the classically described oedema was not reported to be present at initial presentation by the referring veterinary surgeon and was not seen whilst the animal was at the SCPAHFS. This case highlights the fact that whilst hind limb and udder/scrotal oedema occur in typical cases of *M.wenyonii* infection, the absence of oedema cannot rule out infection, as atypical infections do occur. *M.wenyonii* should be considered a differential diagnosis in any cow presenting with anaemia, particularly if there has been a recent reduction in milk yield. If bacteraemia is at a sufficient level, it can be economically diagnosed by taking an EDTA-anticoagulated blood sample and preparing a fresh smear for submission to your local reference laboratory. Blood smears can be examined microscopically for erythrocyte inclusion bodies by a haematologist or clinical pathologist.

Anaemia is an inconsistent finding in previous reports of cattle infected with *M.wenyonii*; when reported, anaemia is most commonly mild to moderate and associated with other clinical signs such as pyrexia, malaise and/or oedema of the hind limbs and udder or scrotum [3, 9, 10, 24, 25]. The case reported here presented with a severe anaemia in the absence of associated clinical signs such as those previously mentioned, which to our knowledge has not yet been reported in a mature animal. Severe anaemia is more commonly reported in splenectomised calves experimentally infected with *M.wenyonii* parasites [26, 27], however there is one recent report of severe anaemia occurring in a naturally infected mature cow, which also presented with hind limb oedema [3]. There are reports of adult cattle co-infected with *M.wenyonii* and other blood parasites such as *Anaplasma* species, *Babesia* species and *Theileria* species presenting with severe haemolytic anaemia [13] and in sheep concurrently infected with *M.ovis* and *Anaplasma ovis* severe haemolytic anaemia has been reported [28]. There was no evidence of concurrent *Anaplasma* or *Babesia* infection in the case reported and *Theileria* species are not present in the United Kingdom.

The case reported was direct Coombs’ test positive, indicating an immune mediated response resulting in haemolysis which to our knowledge has not been reported in cattle infected with *M.wenyonii* before. However, as previously mentioned, an immune-mediated haemolytic process has been implicated in the underlying pathogenesis resulting in anaemia in other species infected with haemotropic *Mycoplasma* species and it is probable that this also occurs in cattle, but until now has not been identified.

Since haemotropic mycoplasmas are uncultivable [19, 20, 29] *Mycoplasma* infection has traditionally been diagnosed solely on the presence of inclusion bodies on blood smears as described previously [24]. Specificity of this test is high however sensitivity can be low [20] due to the lack of a specific stain for *M.wenyonii* on blood smears. In addition, blood smear examination cannot be used to determine which *Mycoplasma* species is involved. Recent advances have enabled the use of 16S rDNA PCR and denaturing gradient gel electrophoresis (DGGE) to more accurately detect and speciate mycoplasmas*,* including haemotropic mycoplasmas. The primer set used in this case report are *Mycoplasma* genus-specific primers [4, 5] and are more commonly used in our laboratory to detect non-haemotropic mycoplasmas.

A volume of 200 μL whole blood in EDTA was sufficient to detect *Mycoplasma* in the first un-treated sample. However, *Mycoplasma* was not detected in 200 μL blood sampled during treatment and immediately prior to euthanasia; positive and negative template controls performed as expected. *Mycoplasma* was however detected in the latter sample when a much larger volume of blood was processed (7 mL). The limits of detection for *Mycoplasma* in bovine blood using this PCR are not known, but 200 μL may be inadequate to detect low *Mycoplasma* loads. Further evaluation of this assay with positive clinical material is needed.

How *M.wenyonii* spreads between cattle is currently unknown but arthropod vectors have been suggested as a possible mode of transmission. Arthropod transmission has been implicated in the spread of *M.haemofelis* between cats [7, 30]. In addition, transmission of *Mycoplasma ovis* by *Culex annulirostris* mosquitoes has been demonstrated in Australia [31]. Extrapolating from this evidence, arthropod vectors could be a possible route of infection in cattle and this is supported by limited Animal Health and Plant Agency (AHPA) (formerly Animal Health and Veterinary Laboratories Agency (AHVLA)) data suggesting the presence of a seasonal incidence in *M.wenyonii* infection in cattle in the United Kingdom [2]. It is also possible that management interventions that result in exposure of uninfected animals to blood from infected animals (e.g. vaccination, ear-tagging, dehorning, castrating etc.) may result in transmission of *M.wenyonii*, as has been suggested in other species [15]. However, neither of these modes of transmission has been proven in cattle. Further work is warranted to determine the mode of transmission of *Mycoplasma wenyonii* in cattle and how this may affect the herd as a whole.

The recommended treatments for *M.wenyonii* infection are tetracyclines, macrolides or fluoroquinolones [2, 29], all of which have readily accessible licensed formulations in the United Kingdom. The use of tetracyclines has been reported in cattle to result in short-term improvement in clinical signs, however treatment is unlikely to shorten the duration of disease [3, 9, 10]. Oxytetracycline and doxycycline have both been used in cats to successfully treat *M.haemofelis* [32] and Lester *et al*. [33] report a case of successful treatment of a dog infected with *M.haemocanis* (formerly *Haemobartonella canis*) with tetracycline [33].

It is possible that prompt treatment with antibiotics may prevent further production losses and spread to other animals in the herd; however, there are no reports of complete cure being achieved in cattle infected with *M.wenyonii* and it is likely that *Mycoplasma wenyonii -*infected cows, in common with other species infected with mycoplasmas*,* remain chronic carriers for the rest of their lives [3, 19]. There is little information available on the effect of chronic haemotropic *Mycoplasma* infection on production [34] and further study is warranted to determine whether a chronic carrier status affects the lifetime production of the infected animal.

## Conclusion

We report this unusual case of *M.wenyonii* infection in a dairy cow. *M.wenyonii* should be considered as a possible differential diagnosis for cattle presenting with anaemia in practice and a blood smear should be performed as part of the initial examination if the cause of anaemia is not immediately obvious. In common with other animals infected with *Mycoplasma* species it is likely that cows remain chronically infected for life; however, there is little evidence as to whether this affects the lifetime productivity of the animal or not. It is therefore warranted to consider treating animals affected by *M.wenyonii* in order to minimise the effects of acute infection and try to maintain the productivity of the animal. In addition to this, prompt treatment of animals affected with *M.wenyonii* also has positive welfare benefits.

## Abbreviations

AHVLA: Animal Health and Veterinary Laboratories Agency
ALKP: Alkaline phosphatase
APHA: Animal and Plant Health Agency
AST: Aspartate aminotransferase
BVD: Bovine Viral Diarrhoea
DGGE: Denaturing gradient gel electrophoresis
GGT: Gamma-glutamyl transferase
GLDH: Glutamate dehydrogenase
IBR: Infectious Bovine Rhinotracheitis
PCR: Polymerase chain reaction
SCPAHFS: Scottish Centre for Production Animal Health and Food Safety
